# The variability of functional MRI brain signal increases in Alzheimer's disease at cardiorespiratory frequencies

**DOI:** 10.1038/s41598-020-77984-1

**Published:** 2020-12-09

**Authors:** Timo Tuovinen, Janne Kananen, Zalan Rajna, Johannes Lieslehto, Vesa Korhonen, Riikka Rytty, Heli Mattila, Niko Huotari, Lauri Raitamaa, Heta Helakari, Ahmed Abou Elseoud, Johanna Krüger, Pierre LeVan, Osmo Tervonen, Juergen Hennig, Anne M. Remes, Maiken Nedergaard, Vesa Kiviniemi

**Affiliations:** 1 Oulu Functional Neuroimaging, Medical Imaging, Physics and Technology, University of Oulu, Oulu, Finland; 2 Medical Research Center, Oulu University Hospital, Oulu, Finland; 3 Center for Machine Vision and Signal Analysis, University of Oulu, Oulu, Finland; 4 Center for Life Course Health Research, University of Oulu, Oulu, Finland; 5 Department of Neurology, Hyvinkää Hospital, Helsinki University Hospital, Hyvinkää, Finland; 6 Department of Diagnostic Radiology, Helsinki University Hospital, Helsinki, Finland; 7 Research Unit of Clinical Neuroscience, Neurology, University of Oulu, Oulu, Finland; 8 Department of Radiology, Medical Physics, Medical Center - University of Freiburg, Faculty of Medicine, University of Freiburg, Freiburg, Germany; 9 Department of Radiology, Cumming School of Medicine, University of Calgary, Calgary, Canada; 10 Department of Paediatrics, Cumming School of Medicine, University of Calgary, Calgary, Canada; 11 Hotchkiss Brain Institute and Alberta Children’s Hospital Research Institute, University of Calgary, Calgary, Canada; 12 Center for Translational Neuromedicine, University of Rochester Medical Center, Rochester, NY USA

**Keywords:** Alzheimer's disease, Diagnostic markers

## Abstract

Biomarkers sensitive to prodromal or early pathophysiological changes in Alzheimer’s disease (AD) symptoms could improve disease detection and enable timely interventions. Changes in brain hemodynamics may be associated with the main clinical AD symptoms. To test this possibility, we measured the variability of blood oxygen level-dependent (BOLD) signal in individuals from three independent datasets (totaling 80 AD patients and 90 controls). We detected a replicable increase in brain BOLD signal variability in the AD populations, which constituted a robust biomarker for clearly differentiating AD cases from controls. Fast BOLD scans showed that the elevated BOLD signal variability in AD arises mainly from cardiovascular brain pulsations. Manifesting in abnormal cerebral perfusion and cerebrospinal fluid convection, present observation presents a mechanism explaining earlier observations of impaired glymphatic clearance associated with AD in humans.

## Introduction

The presence of aggregates of amyloid-beta (Aβ) protein and tau-protein in brain tissue are canonical pathologies of established Alzheimer’s disease (AD)^1,2^. The mechanism ultimately resulting in deposition of these aggregates remains unclear^3^, and the causal relationship between Aβ and tau deposition is debated^4^. The clinical diagnosis of AD is based on a battery of examinations, including neurological and neuropsychological tests, supplemented in recent years by structural MRI of the brain and metabolic imaging with FDG-PET^5,6^. However, the diagnostic accuracy of available approaches is not completely satisfactory^7,8^, and is especially problematic in early or prodromal phases of the disease^9^. In 2018, the National Institute on Aging and Alzheimer’s Association of the United States proposed a shift of the diagnostic paradigm away from clinical symptoms or post mortem findings, towards criteria based on some combination of biomarkers in living persons^10^. While there has been some success in developing diagnostic tests based on analysis of markers in cerebrospinal fluid (CSF)^11,12^, the requirement for invasive sampling is especially onerous in this vulnerable and aged population. An ideal biomarker for diagnosis of incipient Alzheimer’s disease would be minimally invasive and yet highly sensitive to the brain pathology occurring at an early disease stage, thus perhaps enabling timely interventions.

Age, cerebrovascular disease, and hypertension are among the known risk factors for AD^7^. Indeed, aggressive treatment of high blood pressure in prospective studies lowered the risk of conversion from mild cognitive impairment (MCI) to Alzheimer’s dementia^13^. Thus, cardiovascular function bears some relationship with human AD pathophysiology. Cardiovascular pulsations in the brain parenchyma have been shown to drive the recently identified glymphatic clearance system of the brain, which utilizes perivascular spaces as channels to clear soluble proteins and various metabolites into the CSF and thence out of the central nervous system (CNS)^14–16^. Notably in the present context of AD, the glymphatic system is the major pathway responsible for clearance of soluble Aβ from the brain interstitial fluid^14^. Aging, even in the absence of frank atherosclerosis, is associated with reduced elasticity of arterial walls, which leads to attenuation of glymphatic pulsation^17^. Similarly, increasing arterial blood pressure reduces pulsatility of the arterial wall, and reduces the net outflow of CSF along the perivascular spaces^18^. Thus, declining glymphatic function may be a factor in the accumulation of Aβ and other toxic aggregates associated with AD.

**Figure 1:**
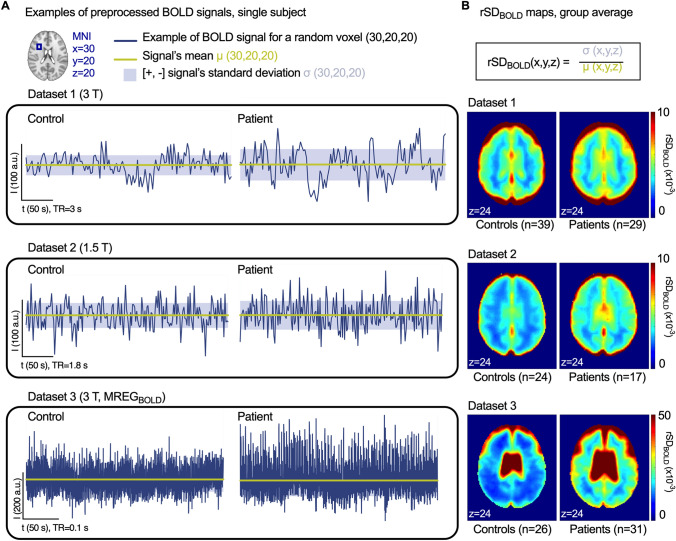
BOLD signal time series and formation of brain signal variability maps (rSD_BOLD_) (**A**) Examples of random single voxel BOLD signals, the signal’s mean and standard deviation for control subjects and AD patients from each dataset. Location of the random region-of-interest in MNI coordinates is shown in the group mean brain maps (**B**). As a measurement of brain signal variability, we calculated rSD_BOLD_ for each voxel and formed 3D maps.

The blood oxygen level-dependent (BOLD) signal in functional MRI (fMRI) has mainly been used to measure very low frequency (VLF) hemodynamic responses to neuronal activity oscillations. However, the BOLD signal is also an indicator of basic underlying vascular and respiratory factors in the brain^19–23^. Recent research has indicated an elevated variability in the BOLD signal, i.e. the relative standard deviation or coefficient of variation of the full band BOLD signal, in brain of AD patients^24,25^. We hypothesized that the physiological brain pulsations driving the glymphatic clearance are altered in AD in a manner manifesting in increased variability in the brain BOLD signal. The objectives of this study were to (I) verify previous findings of altered brain BOLD signal variability in AD; (II) to develop a non-invasive MR-based biomarker for AD without requiring exogenous contrast agents; and (III) to study the physiological origin of the signal alteration. We examined three independent datasets from a total of 80 AD patients and 90 age-matched controls to evaluate brain signal variability reported as the relative standard deviation of the BOLD signal (rSD_BOLD_) (Fig. 1). Using these datasets, we determined the replicability and effectiveness of rSD_BOLD_ in discriminating between AD patients and controls and confirmed the longitudinal repeatability of rSD_BOLD_ for AD detection. Finally, we used ultrafast functional MRI scanning in the third dataset (MREG_BOLD_) with physiological data of cardiopulmonary function to test our hypothesis that increased BOLD signal variability is more related to physiological mechanisms driving the glymphatic brain clearance than an indicator of VLF fluctuations of the BOLD signal arising from perturbed neuronal activity.

## Results

We used data from a sample of 80 AD patients and 90 healthy controls compiled from three independent datasets (two local and one from International Alzheimer’s disease Neuroimaging Initiative ADNI), who were imaged without exogenous contrast agents using either conventional or fast fMRI (Table 1). After conventional preprocessing and quality control of the imaging data, BOLD signal sequences from 89 healthy controls and 77 AD patients were eligible for analysis. Demographics and clinical data of the included participants are summarized in Table 2. We used the relative standard deviation of the BOLD signal (rSD_BOLD_) to measure brain signal variability^26–28^ (Fig. 1). rSD_BOLD_ is also known as the coefficient of temporal variation (CV), where a higher CV value equals greater variability of amplitude of the BOLD signal.

**Table 1:** Imaging parameters for functional data**.**

	Dataset 1	Dataset 2	Dataset 3
Scanner	Philips (13 sites)	GE Signa HDx	Siemens Skyra
Field strength (T)	3	1.5	3
Sequence	EPI	EPI	MREG, 3D spiral single shot
TR (ms)	3000	1800	100
TE (ms)	30	40	36
Duration (volumes/time)	140/7 min	202/6 min 4 s	2961/5 min
FA (deg)	80	90	25
Voxel size (mm)	4 × 4 × 4	3 × 3 × 3	3 × 3 × 3
Slice thickness (mm)	3.3	4	3
Matrix size	64 × 64	64 × 64	64 × 64

TR = Repetition time. TE = Echo time. FA = Flip angle.

**Table 2:** Participant demographics.

	Participants	Age (years)	Female	Disease duration (years)	MMSE	Average fMRI datasets per participant
**Dataset 1 (ADNI)**						
AD patients	29	71.7 ± 6.5	14 (48%)	NC	21.8 ± 3.6 * [12–28]	2.1 [1–3]
Controls	39	72.7 ± 4.3	25 (62%)	-	29.1 ± 1.3 [24–30]	2.2 [1–3]
**Dataset 2 (local)**						
AD patients	17	60 ± 5.4	11(65%)	2.6 ± 1.3	22.9 ± 2.6 * [18–27]	1
Controls	24	60.0 ± 5.1	12 (50%)	-	29.0 ± 1.1 [26–30]	1
**Dataset 3 (local)**						
AD patients	31	60.5 ± 4.8 *	18 (58%)	3.4 ± 2.3	22.3 ± 6.3 * [10–30]	1
Controls	26	57.3 ± 5.7	16 (62%)	-	28.6 ± 1.2 * [25–30]	1

Descriptive demographic characteristics of the groups. Values represent mean ± SD or N (%). [Range]. NC = Not collected, multiple datasets. MMSE = Mini Mental State Examination (maximum total score is 30). * patients versus controls, where P < 0.05.

### Brain signal variability is increased in AD

**Figure 2:**
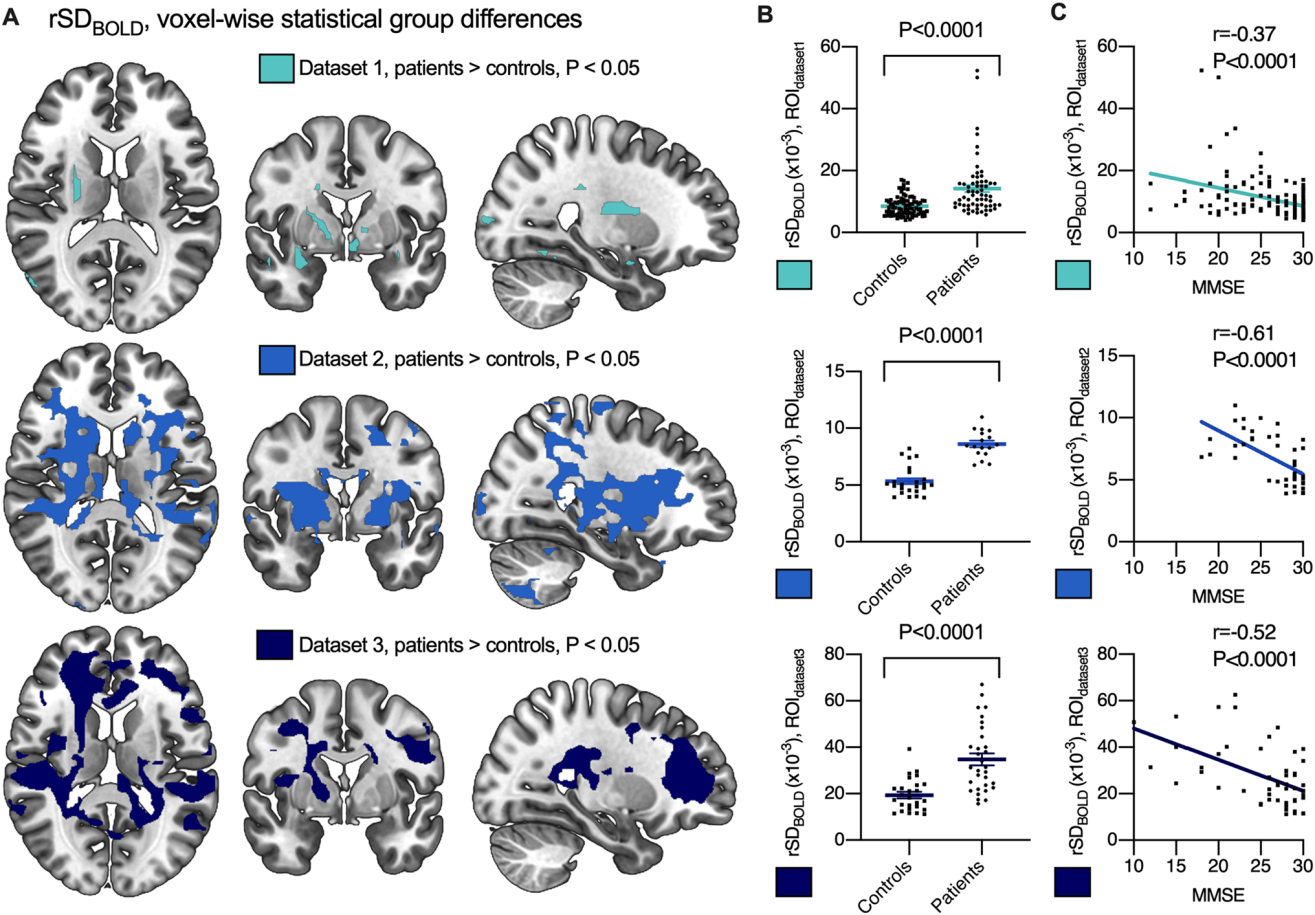
Brain BOLD signal variability in patients with AD compared with controls. Differences in rSD_BOLD_ according to whole-brain voxel-wise analyses (**A**). Maps represent group-level differences where rSD_BOLD_ is higher in Alzheimer’s disease patients (P < 0.05, family wise error corrected). These maps are used as region-of-interests (ROI_dataset1_, ROI_dataset2_, ROI_dataset3_) in further analysis. (**B**) chart shows the mean ± standard error of the mean rSD_BOLD_ values to group, extracted from each ROI_dataset1-3_ e.g. significant clusters determined in **A**. (**C)** shows correlation between rSD_BOLD_ and Mini-Mental State Examination (MMSE).

In whole brain voxel-wise analysis, AD patients showed increased rSD_BOLD_ (P < 0.05, corrected for multiple comparisons, and with head motion parameters used as a regressor) in clusters of voxels distributed around the basal ganglia and in the white matter around lateral ventricles (Fig. 2 and S1). These clusters were defined as regions-of-interest (ROIs) for further analysis (ROI_dataset1-3_). There were no regions with significantly higher rSD_BOLD_ in controls relative to the patients. We further used voxel-based morphometry (VBM) to compare patterns of neurodegeneration with the regional rSD_BOLD_ changes. The extent of gray matter atrophy in the AD group was in line with the previous literature^5,29^ and had only partial overlap with the clusters of increased rSD_BOLD_ (Figs. S1, S2).

### Brain signal variability increase is associated with declined MMSE

The Mini-Mental State Examination (MMSE) was used to test for cognitive impairment in the participants (Table 2). MMSE scores range from 0 to 30, with a score of 10 to 26 indicating mild-to-moderate cognitive impairment ^30^. There was negative correlation with MMSE and average rSD_BOLD_ within ROIs from each of the three datasets (r = − 0.37 to − 0.61; P < 0.0001), meaning that increased BOLD signal variability was associated with lower cognitive function scores (Fig. 2C).

### Replicability of brain signal variability increase

First, present results replicated previous findings of increased BOLD signal variability in AD patients compared to controls^24,25^. We next assessed the anatomical concordance of the regions of increased rSD_BOLD_ in the three independent AD datasets in this study. There were altogether 948 common voxels (7.6 cm^3^) that showed significantly increased rSD_BOLD_ in all three datasets. The anatomical patterns of statistically significant changes in rSD_BOLD_ were markedly alike in all three datasets, despite the use of different imaging setups and parameters (Table 1, Figs. S1 and S2). Notably, scanning protocols differed with respect to sampling rate (repetition time, TR) (Fig. 1). The spatial extent of the rSD_BOLD_ increases in AD patients versus controls was larger in dataset 2 than in dataset 1, and larger still in dataset 3 (MREG_BOLD_), which matched the rank order to the increasing sampling rates, i.e. faster temporal scanning. That association shows that the temporal resolution of the data is an important factor for the detection of AD-dependent rSD_BOLD_ alterations.

### Accuracy and repeatability of brain signal variability in discriminating AD patients from controls

**Figure 3:**
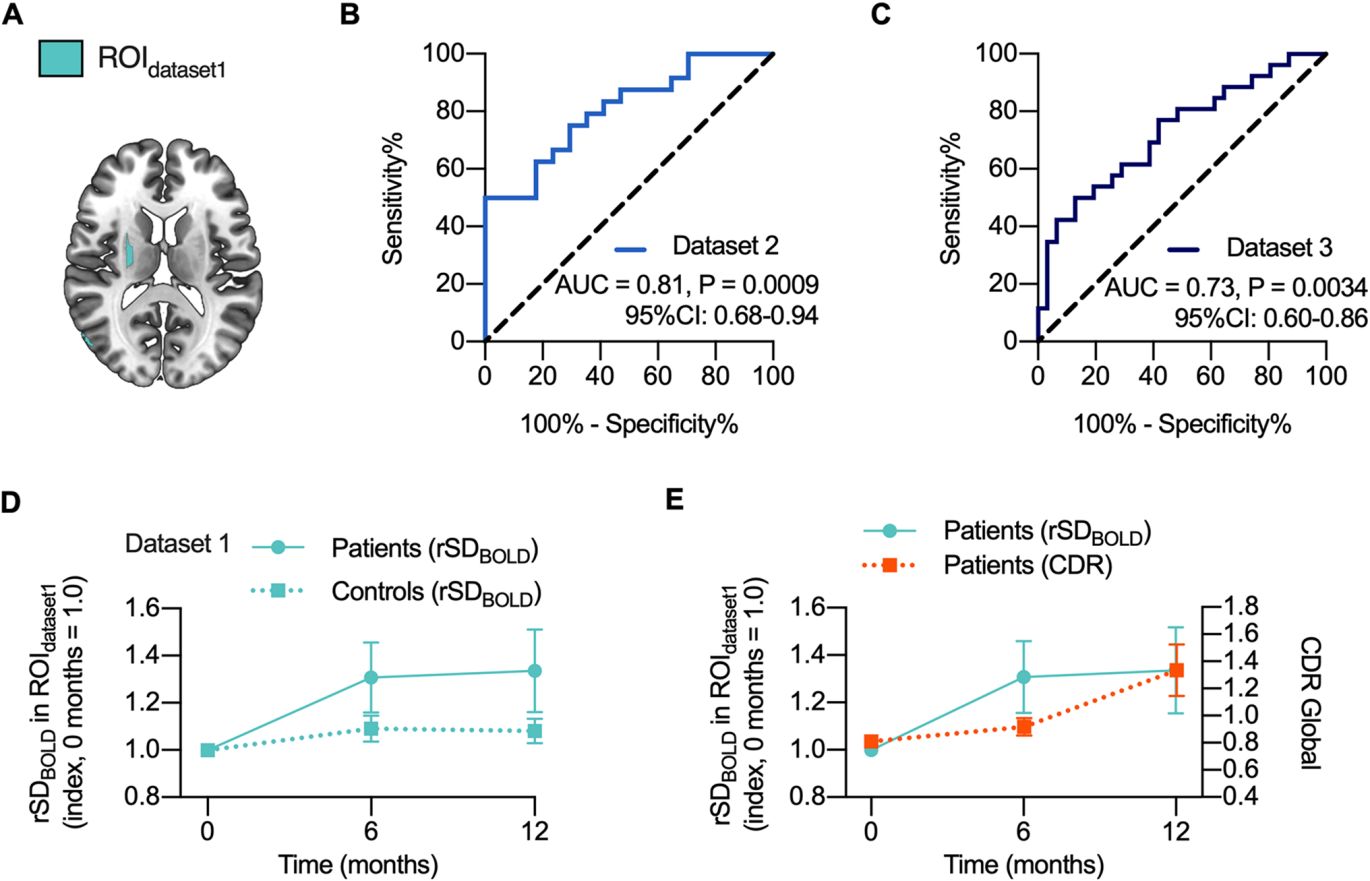
Accuracy and repeatability. (**A**) Region-of-interest (ROI_dataset1_) was defined by significant clusters in dataset 1 (c.f. Figure 2A). Receiver operating characteristic (ROC) curves and area-under-curve (AUC) for differential diagnosis was based on mean rSD_BOLD_ values in datasets 2 (**B**) and 3 (**C**) within this ROI_dataset1_. Confidence intervals and statistical significance are also shown. (**D**) Within-individual changes in the average rSD_BOLD_ in ROI_dataset1_ over time after baseline imaging (0 months) in AD patients and controls in dataset 1. There was statistical difference between the two groups at both 6 and 12 months. Data represents the mean ± standard error of the mean. Mixed-effect analysis significance between groups p < 0.035 and between timepoints p < 0.0054 corrected for multiple comparison. Predicted mean increase was in AD 21% and in controls 5%. (**E**) presents indexed rSD_BOLD_ change and Clinical Dementia Rating (CDR) global values in follow up in patients.

To our knowledge, this is the first study to analyze diagnostic accuracy and repeatability of increased BOLD signal variability in AD. Receiver operating characteristic (ROC) curve and the area under the ROC curve (AUC) were calculated to establish the feasibility of using rSD_BOLD_ as a biomarker for AD. Dataset 2 (AUC = 0.81, P = 0.0009) and dataset 3 (AUC = 0.73, P = 0.0034) both demonstrated good effectiveness of the rSD_BOLD_ values within ROI_dataset1_ in discriminating between AD patients and controls (Fig. 3A-C).

To analyze the repeatability and possible effect of disease progression of these findings, we scrutinized the follow-up data in the dataset 1. In the control group average rSD_BOLD_ values within ROI_dataset1_ were relatively stable over a 12-month period (+ 8% on average, not significant) (Fig. 3D), thus indicating good repeatability. Importantly, the rSD_BOLD_ increased (+ 32% on average, P = 0.0054) *only* in the AD group (P = 0.035), while Clinical Dementia Rating (CDR) also increased in the patient group, denoting greater cognitive impairment (Fig. 3E), thus indicating a relationship between rSD_BOLD_ increases with disease progression.

### Increased brain signal variability in cardiac and respiratory frequencies

We next asked whether BOLD signal variability is increased in AD patients due to changes in their hemodynamic responses to neuronal activity oscillations (at very low frequencies, VLF) or rather due to other fast physiological components of the BOLD signal, namely cardiac and respiratory pulsations. Previous pioneering work along these lines^24,25^, as well as the datasets 1 and 2 in this study, employed relatively slow sampling rates (TR = 1.8–3.0 s, equivalent to 0.33–0.56 Hz). Thus, any signal with frequency content higher than the corresponding Nyquist rate would have been be aliased into the BOLD signal, which is here the case for cardiac (0.8–1.65 Hz) and parts of the respiratory (0.15–0.4 Hz) signals.

**Figure 4:**
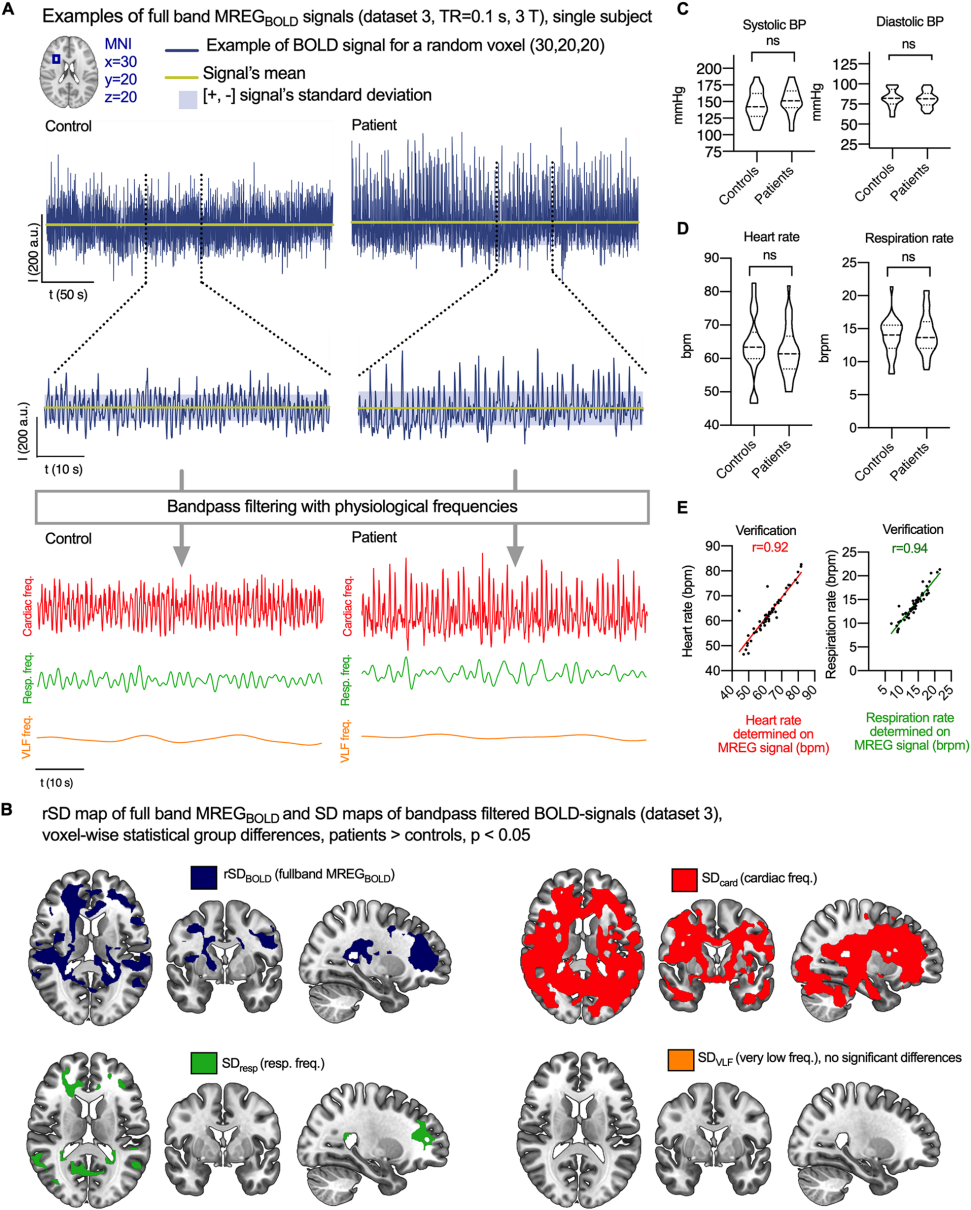
Physiological source mapping. (**A**) MREG_BOLD_ signal is imaged with fast sampling rate (TR = 0.1 s). A representative 60 s clip is shown for the purposes of illustration. MREG_BOLD_ signal is bandpass filtered to physiological frequencies to study the cardiac, respiratory and VLF parts of the signal. (**B**) Differences in rSD_BOLD_ and SD of bandpass filtered signals according to whole-brain voxel-wise analyses. Maps represent group-level differences where SD is higher in Alzheimer’s disease patients (P < 0.05, family wise error corrected). (**C**) Blood pressure (BP) while supine just prior to entering the MR scanner. (**D**) Heart and respiration rates from peripheral pulse oximeter and respiration belt, respectively. (**E**) Heart and respiration rates determined on MREG signal and their correlation to peripheral signals. NS = not significant.

Fast fMRI sequences enable the detection of physiological oscillations that propagate from cardiac and respiratory pulsations within their respective frequency sub-bands^22,31,32^. We used an ultra-fast fMRI sequence (MREG_BOLD_, dataset 3) sampled with TR 0.1 s, corresponding to ten complete brain acquisitions per second, to examine the variation within different frequency bands of the BOLD signal (Fig. 4A). We then calculated SD maps of the MREG_BOLD_ with bandpass filtration at physiological frequencies, aiming to discover the physiological source of increased rSD_BOLD_ in the AD group. Much as with rSD_BOLD_ maps of full band signal described above, we calculated the whole brain voxel-wise analysis of SD maps of bandpass-filtered MREG_BOLD_ for the AD and control groups (Fig. 4B).

We then compared the voxel-wise group-level difference between the AD and control groups in rSD maps of full band MREG_BOLD_ and in SD maps of cardiac (SD_card_), respiratory (SD_resp_), and VLF (SD_VLF_) band data. This showed that rSD maps of the full band and SD maps of cardiac and respiratory parts of the signal overlapped spatially. Moreover, the SD_card_ showed widespread increases extending beyond the corresponding findings for full band rSD_BOLD_ in the AD group (Fig. 4B). Most areas adjacent to periventricular structures showed increased SD_card_, especially in the posterior brain regions. The increased SD_card_ extended down from somatosensory areas and encompassed most of the periventricular white matter. Increased SD_card_ could also be discerned in the bilateral basal ganglia of AD patients, but there were no such changes in frontal, insular and visual cortex (Fig. 4). All the regions with elevated respiratory SD_resp_ overlapped with regions with increased SD_card_.

The SD_resp_ maps indicated group differences in bilateral frontal and temporoparietal areas, left sensorimotor cortex, medial frontal and temporal gyri. Notably, the anterior and posterior corpus callosum and bilateral angular gyri of the default mode network both also had increased SD_resp_ in the AD group. Between-group voxel-wise differences in SD_VLF_ maps did not attain statistical significance (P = 0.06–0.1).

### No difference in blood pressure, heart or respiration rate between patients and controls

To verify the nature of cardiac and respiratory signals, we used simultaneous within-subject cardio-respiratory data derived from peripheral heart rate pulse oximeter and respiratory belt measurements in dataset 3. Importantly, there were no between-group differences in heart or respiratory rate nor in the blood pressure, which was measured twice just before the scanning (Fig. 4C-D). We calculated average heart and respiratory rate from the MREG_BOLD_ signal for each subject. These results had a strong positive correlation (r = 0.92–0.94, P < 0.0001) with the physiological verification data (Fig. 4E).

## Discussion

Using three independent fMRI datasets, we confirmed previous findings that brain signal variability (rSD_BOLD_) is increased in AD^24,25^. ROC curve analysis results indicated that the rSD_BOLD_ has good precision for the differentiation of AD patients and controls. Indeed, the ROC AUC (0.73–0.81) by present methods is comparable to that reported in various invasive biomarker studies^6,8,12^. Longitudinal follow-up data from the ADNI study showed that the rSD_BOLD_ measure is repeatable in controls but increases with time in individual AD patients scanned with one-year follow-up. This indicates an association between elevated rSD_BOLD_ and disease progression. Increased rSD_BOLD_ also correlated with lower MMSE values in all three datasets, denoting a general association with cognitive dysfunction. Our findings in the dataset with ultrafast MREG_BOLD_ and simultaneous within-subject cardio-respiratory data shows that the physiological BOLD signal variability in AD patients originates predominantly from variability of cardiac pulsatility, and to a lesser degree respiratory signal variability.

Recent research indicates that the glymphatic brain clearance driven by physiological brain pulsations might be impaired in AD^18,33,34^. The glymphatic influx of CSF tracers is driven by arterial wall pulsations driven by the cardiac cycle^18^, while other functional neuroimaging studies indicate that both the cardiovascular and the respiratory pulsations drive CSF convection^22,32,35^. Our present use of fast MREG_BOLD_ signals sampled at 10 Hz in dataset 3 enables exact separation of the cardiorespiratory pulsation from the low frequency BOLD signal without aliasing^36^. We thus find that the increased brain BOLD signal variability in the AD group is mainly derived from or driven by cardiac frequency brain pulsations, which are also understood to be the main driver for glymphatic paravascular fluid transport^18^. The respiratory pulsations appear to play a lesser role in provoking the BOLD signal variability increase often detected in AD populations.

The peripheral cardiorespiratory measurements in the subjects from dataset 3 indicate that general features of cardiorespiratory physiology are insufficient to explain the increased intracranial BOLD signal variability in AD, as we note that the neither the average cardiorespiratory rates nor the blood pressure differed between the control and AD groups in that study. On the other hand, the cardiovascular pulse propagation *inside* the brain parenchyma has been found to be more variable in AD^37^. Although the VLF fluctuations are normally the dominant source of variance in BOLD signal, we find that in AD, the main driver for this variability was brain pulsations arising from cardiac frequency.

There are some strengths and limitations that should be considered in the interpretation of the present results. First, there is a certain lack of standards in methodology and terminology in this area of research. A common issue in fMRI scanning has been the dependence of the results on scanner and imaging parameters. However, the concordance of present rSD_BOLD_ results from three independent datasets seems to indicate a consistent pattern of AD-related changes, despite quite substantial differences in the different vendor scanners and scanning parameters. The rSD_BOLD_ results are robust for a broad range of BOLD image sampling rates, this consistently indicating a source in physiological cardiopulmonary pulsations. Since results from conventional and ultrafast fMRI agree, both methods seem applicable for diagnostic purposes. However, larger replication samples with longitudinal fMRI measurements shall be required. Prospective studies of preclinical or prodromal AD with fMRI should serve to establish the diagnostic usefulness of present methods. As dementia is a clinical syndrome with variable manifestations and is often accompanied by neuropsychiatric and behavioral problems, further work with different patient groups is required to establish the viability of this method as a differential diagnosis tool ^29^.

In conclusion, we detected increased physiological BOLD signal variability in AD. The new index of rSD_BOLD_ may offer a non-invasive biomarker for diagnosis of AD without exogenous requiring CSF sampling or scanning in conjunction with MR contrast agents or nuclear imaging. Our results implicate altered intracranial cardiorespiratory pulsation as the driver for increased rSD_BOLD_, which may help to tie together glymphatic clearance and pathophysiological mechanisms underlying AD. Future studies should investigate how rSD_BOLD_ is changed in other neuropathological conditions and during the course of MCI in a longitudinal study design with large patient cohorts. In addition, studies revealing a mechanistic relation between rSD_BOLD_ and other biomarkers such as Aβ and tau are required to understand the bigger picture of the underlying AD pathogenesis.

## Materials and methods

### Study design

The objectives of this study were to (I) verify previous findings of increased BOLD signal variability (rSD_BOLD_) in AD, (II) to develop a non-invasive imaging biomarker for AD without requirement for exogenous MR contrast agents or ionizing radiation exposure; and (III) to study the physiological origin of the increased rSD_BOLD_. We used a sample of 80 AD patients and 90 healthy controls derived from three independent datasets, one being the publicly available international multisite ADNI study (dataset 1), and two having been acquired at our institute (datasets 2–3). No power analysis was performed before the study; the dataset sizes were in line with previous experiments using functional imaging in patients with AD. Data collection and further analyses were not performed blind to the conditions of the experiment. However, preprocessing of neuroimaging data included standard automated analytic pipelines, which were agnostic to the diagnostic and demographic characteristics of the data.

### Participants

All the patients of the AD groups met the NINCDS-ADRDA (National Institute of Neurological and Communicative Disorders and Stroke and the Alzheimer’s Disease and Related Disorders Association) criteria for AD^5^. The control subjects were interviewed, and Mini-Mental State Examination (MMSE) and Beck’s Depression Inventory (BDI) were performed to exclude any cases of dementia. Any psychiatric or neurological disorders or medications affecting the central nervous system were exclusion criteria for the control group.

As per ADNI protocols, all procedures performed in studies involving human participants were in accordance with the ethical standards of the institutional and/or national research committee and with the 1964 Helsinki declaration and its later amendments or comparable ethical standards. More details can be found at http://adni.loni.usc.edu. As per local data, written informed consent was obtained from all the participants or their legal guardians prior to scanning. The study was approved by the Regional Ethics Committee of the Northern Ostrobothnia Hospital District. Research was conducted accordance with Helsinki declaration**.**

**Dataset 1** used in the preparation of this article was obtained from the ADNI database (http://adni.loni.usc.edu). ADNI was launched in 2003 as a public–private partnership, led by Principal Investigator Michael W. Weiner, MD. The primary goal of ADNI has been to test whether serial magnetic resonance imaging (MRI), positron emission tomography (PET), other biological markers, and clinical and neuropsychological assessment can be combined to measure the progression of mild cognitive impairment (MCI) and early AD. For up-to-date information, see http://www.adni-info.org. We selected ADNI-2 participants in whom resting state fMRI and preprocessed anatomical scans had been obtained within the first year of their participation in the study. One-year follow-up data was available from dataset 1. All the patients of the **datasets 2–3** had been examined by experienced neurologists specialized in memory disorders at the outpatient memory clinic of the Department of Neurology of Oulu University Hospital in Finland. The Finish AD patients underwent a battery of examinations, including clinical and neurological examinations, screening laboratory tests, a neuropsychological examination and both structural and functional MRI of the brain. As required for clinical workup, CSF analyses of the biomarkers Aβ42, tau and phospho-tau and/or functional neuroimaging by fluorodeoxyglucose positron emission tomography (FDG-PET) had been performed on the patients to confirm their diagnosis. All of the AD group patients met the current diagnostic criteria for AD^5^. The control subjects were interviewed, and Mini-Mental State Examination (MMSE) and Beck’s Depression Inventory (BDI) were performed to screen for any individuals with memory deficits or depression. Any psychiatric or neurological disorders or use if medications affecting the central nervous system were exclusion criteria for the control group. There was no follow-up imaging data for the subjects in datasets 2–3.

After preprocessing and quality control of the three datasets, one control and three AD patients were excluded from the study owing to excessive head motion or other artifacts in the data, and one individual with AD was excluded because of the incidental finding of a brain tumor. A total of 89 healthy controls and 77 AD patients were eligible for analysis. Demographics and clinical data of the included participants are summarized in Table 2. There were no differences between the groups for age and sex demographics in datasets 1 and 2. In dataset 3, the AD patients were of slightly greater mean age (P = 0.02).

### Imaging data

Each subject was imaged for both functional and structural MRI. Details of the scanning parameters for the anatomical scans are shown in Supplement Table 1. Imaging parameters for functional MRI are shown in Table 1. Notably, the three scanning setups differed with respect to sampling rate (repetition time, TR) and magnetic field strength. Datasets 1 and 2 were acquired using typical EPI-BOLD sequence with 3 T and 1.5 T scanners, respectively. As noted above, dataset 1 also included follow-up imaging up at baseline and at six and 12 months.

FMRI from dataset 3 was acquired with magnetic resonance encephalography (MREG) sequence, which is a three-dimensional (3D) spiral, single shot sequence that undersamples 3D k-space in a spiral trajectory for faster imaging^38^. The method samples the BOLD signal at 10 Hz frequency, thus 20–25 times faster than in conventional fMRI, such as used in datasets 1 and 2. The high sampling rate of the MREG sequence is crucial to evaluate phenomena in the frequency range of cardiovascular pulsations. While there are a few alternatives for fast sampled fMRI sequences, MREG has been proven suitable to measure physiological pulsations^32,37^.

### Physiological data

For dataset 3 we also collected peripheral heart rate measured using a pulse oximeter (SpO_2_ signals from fingertip) and respiratory rate measured using the scanner’s respiratory belt. Also, the blood pressure was measured with subjects in a supine position just prior to imaging using GE Datex-Ohmeda Aestiva5/MRI compatible anesthesia monitor.

### Preprocessing of imaging data

Preprocessing for all three datasets was conducted using the Oxford Centre for Functional MRI of the Brain Software Library 5.0 (FSL 5.0.11, http://www.fmrib.ox.ac.uk/fsl)^39^ exactly as described in^27^. **For fMRI data, the pipeline included** head motion correction, brain extraction, spatial smoothing, and high-pass temporal filtering. Multi-resolution affine co-registration within FSL FLIRT software was used to co-register the mean, non-smoothed fMRI and structural maps of corresponding subjects (as described below), and to co-register these volumes to the Montreal Neurological Institute’s (MNI152) standard space template. **Motion analysis**: From head motion correction parameters (MCFLIRT), we extracted subject-wise absolute displacement vectors (in mm), which describe the amount of movement in all directions over the entire scan as a marker of gross head motion. Also, relative displacement vectors were extracted, as a marker of motion between each EPI volume. Both vectors were also averaged across volumes to get the mean values. **Structural data and gray matter atrophy (GM) maps:** After visual inspection by an experienced neuroradiologist, structural data was analyzed with FSL-VBM (http://fsl.fmrib.ox.ac.uk/fsl/fslwiki/FSLVBM), an optimized VBM protocol executed with FSL tools. First, structural images were skull-stripped and the gray matter was segmented before being registered to the MNI 152 standard space using non-linear plastic registration. The resulting images were averaged and flipped along the x-axis to create a left–right symmetric, study-specific grey matter template. Second, all native grey matter images were non-linearly registered to this study-specific template and "modulated" to correct for local expansion (or contraction) due to the non-linear component of the spatial transformation. The modulated grey matter images were then smoothed with an isotropic Gaussian kernel with a sigma of 3 mm.

### Brain signal variability maps (rSD_BOLD_)

For each participant, a map of rSD_BOLD_ was computed as the relative standard deviation of the BOLD timeseries at each voxel from preprocessed functional MRI data:

1 $$ rSD_{BOLD} = \frac{{\upsigma ({\text{X}}_{BOLD} )}}{{\upmu ({\text{X}}_{BOLD} )}} $$

where X_BOLD_ is voxel time series, σ is the standard deviation and μ is the mean. Representative examples of preprocessed BOLD signals, their standard deviation (SD), mean and the calculated group average rSD_BOLD_ maps are shown in Fig. 1 for all three datasets.

### Physiological source mapping (SD_card_, SD_resp_ and SD_VLF_)

To study the physiological source of BOLD signal variability, we bandpass filtered the critically sampled MREG_BOLD_ signal (dataset 3) to cardiac (> 0.6 Hz), respiratory (0.1–0.6 Hz) and very low frequencies (VLF, 0.008—0.1 Hz), using AFNI 3dTProject^40^. For each participant, SD maps of cardiac, respiratory and VLF frequency signals were computed as the standard deviation of the filtered MREG_BOLD_ timeseries at each voxel. Since bandpass filtering removes the signal mean, we here reported standard deviation instead of rSD.

To verify reliability of these signals, average heart and respiratory rates were determined using Fast Fourier Transform from whole brain full band MREG signals. We calculated Pearson’s correlation coefficients between these measurements and peripheral heart and respiratory rates measured by the pulse oximeter and respiration belt.

### Statistical analysis

All statistical analyses were performed using the GraphPad Prism 8, unless otherwise stated. AD patients were compared to controls in the group-level analysis within each dataset. All results were examined at a P < 0.05 significance level, unless otherwise stated. The χ2-test was used to calculate P-values for categorical variables and the t-test for continuous variables. **Voxel-level statistical analysis of imaging data:** To examine differences in AD patients versus controls, between-group contrast comparisons of the various parametric maps (GM, rSD_BOLD_, SD_card_, SD_resp_ and SD_VLF_) were statistically tested using permutation-based nonparametric testing incorporating threshold-free cluster enhancement (TFCE) and correction for multiple comparisons implemented in the FSL randomise tool with 10,000 random permutations. In functional data analysis (rSD_BOLD_ and SD maps), relative motion parameters were used as regressors due to the small difference between groups in dataset 1 (Table S2). **Region-of-interests (ROIs)**: Statistically significant differences between groups in voxel-level analysis were also used to define region-of-interest (ROI) segments for further analysis. For example, ROI_dataset1_ is defined as the set of voxels with statistically significantly increased rSD_BOLD_ values in the AD group from dataset 1. **Receiver operating characteristic (ROC)** curve and the area under the ROC curve (AUC) were calculated to estimate the feasibility of using rSD_BOLD_ as a potential biomarker for AD. We plotted ROC curves to evaluate whether rSD_BOLD_ could serve to separate healthy controls from patients in datasets 2 and 3. The mean rSD_BOLD_ for each subject was calculated using ROI_dataset1_ (Fig. 2A and 3A), and AUC was calculated as a measure of classification accuracy. The bootstrap approach was used to estimate the 95% confidence interval of AUC. **Follow-up data** in dataset 1 were used to estimate the repeatability and the effect of the disease progression on rSD_BOLD_. We calculated average rSD_BOLD_ within ROI_dataset1_ for each subject in dataset 1 and plotted this as a function of time. Repeated measures mixed-effect analysis was performed on the follow-up data. Clinical Dementia Rating (CDR) was used as a clinical marker for disease progression to follow-up in the AD group. **Spatial correlation analysis:** To analyze the similarity of the results of different modality voxel-level statistical analysis, we performed spatial cross-correlation correlation of P-value maps of different modalities (rSD_BOLD_, GM and SD) using the fslcc command of FSL. **Visualization:** Most of the data was plotted using GraphPad Prism 8. Functional MRI data was plotted using Matlab (Fig. 1) or MRIcroGL (Fig. 2–4, https://www.mccauslandcenter.sc.edu/mricrogl/).

## Supplementary information

Supplementary material 1

## Data Availability

The data that support the findings of this study are available from the corresponding author, upon reasonable request.
