# Supplementary material for: The variability of functional MRI brain signal increases in Alzheimer's disease at cardiorespiratory frequencies
Source: Sci Rep. 2020 Dec 9;10:21559. doi: 10.1038/s41598-020-77984-1 (PMC7726142; doi:10.1038/s41598-020-77984-1)
Supplement: Supplementary file 1 — Supplementary material 1 [file 41598_2020_77984_MOESM1_ESM.pdf]

The variability of functional MRI brain signal increases in Alzheimer's disease at cardiorespiratory frequencies

Timo Tuovinen,[1,2*] Janne Kananen,[1,2] Zalan Rajna,[1,3] Johannes Lieslehto,[4] Vesa Korhonen,[1,2] Riikka Rytty,[1,5] Heli Mattila,[1,2] Niko Huotari,[1,2] Lauri Raitamaa,[1,2] Heta Helakari,[1,2] Ahmed Abou Elseoud,[6] Johanna Krüger,[2,7] Pierre LeVan,[8-11] Osmo Tervonen,[1,2] Juergen Hennig,[8] Anne M. Remes,[2,7] Maiken Nedergaard,[12] Vesa Kiviniemi[1,2,*] for the Alzheimer's Disease Neuroimaging Initiative†

[1]Oulu Functional Neuroimaging, Medical Imaging, Physics and Technology, University of Oulu, Oulu, Finland.

[2]Medical Research Center, Oulu University Hospital, Oulu, Finland.

[3]Center for Machine Vision and Signal Analysis, University of Oulu, Finland

[4]Center for Life Course Health Research, University of Oulu, Oulu, Finland

[5]Department of Neurology, Hyvinkää Hospital, Helsinki University Hospital, Hyvinkää, Finland.

[6]Department of Diagnostic Radiology, Helsinki University Hospital, Helsinki, Finland.

[7]Research Unit of Clinical Neuroscience, Neurology, University of Oulu, Oulu, Finland

[8]Department of Radiology, Medical Physics, Medical Center - University of Freiburg, Faculty of Medicine, University of Freiburg, Freiburg, Germany

[9]Department of Radiology, Cumming School of Medicine, University of Calgary, Calgary, Canada

[10]Department of Paediatrics, Cumming School of Medicine, University of Calgary, Calgary, Canada

[11]Hotchkiss Brain Institute and Alberta Children's Hospital Research Institute, University of Calgary, Calgary, Canada

[12]Center for Translational Neuromedicine, University of Rochester Medical Center, NY, USA

* Corresponding authors:

MD Timo Tuovinen timo.tuovinen@oulu.fi and professor Vesa Kiviniemi, vesa.kiviniemi@oulu.fi

† Data used in preparation of this article were obtained from the Alzheimer's Disease Neuroimaging Initiative (ADNI) database (adni.loni.usc.edu). As such, the investigators within the ADNI contributed to the design and implementation of ADNI and/or provided data but did not participate in analysis or writing of this report. A complete listing of ADNI investigators can be found at: http://adni.loni.usc.edu/wp-content/uploads/how_to_apply/ADNI_Acknowledgement_List.pdf

Supplementary Materials

Fig. S1. Group-level differences in rSD_{BOLD} and gray matter (GM) maps according to voxel-wise analyses.

Fig. S2. Spatial correlation coefficients (fslcc) between voxel-wise group level difference P-value maps.

Table S1. Structural imaging parameters.

Table S2. Motion parameters.

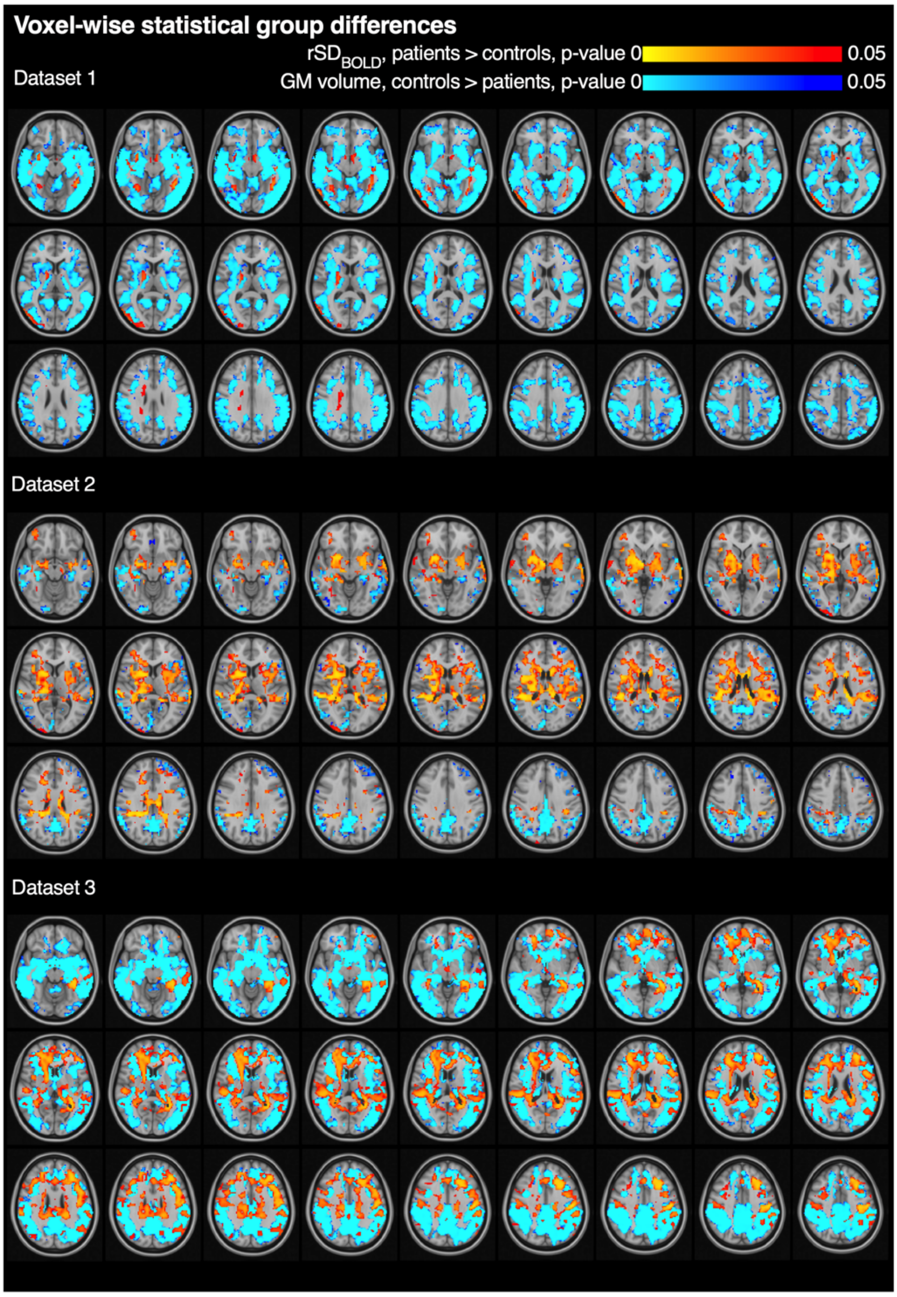

Fig. S1. Group-level differences in rSD$_{BOLD}$ and gray matter (GM) maps according to voxel-wise analyses. rSD$_{BOLD}$ higher in AD patients compared to controls (marked with yellow-red). GM lower in AD patients compared to controls (marked with blue). Data are presented as P-value maps (family wise error corrected, $P<0.05$).

		rSD_{BOLD}			GM			SD maps (dataset 3)		
		Dataset 1	Dataset 2	Dataset 3	Dataset 1	Dataset 2	Dataset 3	SD_{card}	SD_{resp}	SD_{VLF}
rSD_{BOLD}	Dataset 1	1	0,42	0,43	0,39	0,29	0,35	0,53	0,39	0,47
	Dataset 2		1	0,56	0,45	0,27	0,44	0,61	0,47	0,55
	Dataset 3			1	0,46	0,38	0,53	0,78	0,8	0,81
GM	Dataset 1				1	0,46	0,63	0,59	0,44	0,46
	Dataset 2					1	0,49	0,46	0,37	0,34
	Dataset 3						1	0,61	0,49	0,49
SD maps	SD_{card}							1	0,78	0,77
	SD_{resp}								1	0,76
	SD_{VLF}									1

Fig. S2. Spatial correlation coefficients (fslcc) between voxel-wise group level difference p-value maps. To analyze the similarity of group-level differences between different modalities, we used fslcc-tool to calculate spatial correlation coefficient (r) between different thresholded p-value maps ($P<0.05$). On average highest cc were between different SD maps (average r=0.77), then between rSD_{BOLD} - SD maps (average r=0.60), then between GM maps in each dataset (GM maps – GM maps; average r=0.53), then rSD_{BOLD} maps between different datasets (rSD_{BOLD} - rSD_{BOLD}; average r=0.47) and GM maps-SD maps (average r=0.60). Lowest average r was between rSD_{BOLD} - GM maps (average r=0.40).

	**Dataset 1**	**Dataset 2**	**Dataset 3**
Sequence	3D MPRAGE	3D FSPGR BRAVO	3D MPRAGE
TR (ms)	7	12.1	100
TE (ms)	3	5.2	36
FA (deg)	9	20	25
Slice thickness (mm)	1.2	1.0	0.9
Matrix size	256x256x170	256x256 (i.e. 1 mm^3)	64x64x64
FOV (cm)	24	24	24

Table S1. Structural imaging parameters. TR =Repetition time. TE = Echo time. FA = Flip angle. FOV=field of view.

	**Dataset 1**		**Dataset 2**		**Dataset 3**	
	Absolute	Relative	Absolute	Relative	Absolute	Relative
Patients	0.27±0.26	0.18±0.13	0.25±0.12	0.08±0.04	0.24±0.14	0.02±0.03
Controls	0.20±0.14	0.14±0.08	0.23±0.10	0.06±0.03	0.18±0.14	0.02±0.02
t-test	ns	0.036	ns	ns	ns	ns

Table S2. Motion parameters. Head motion correction parameters (MCFLIRT) we extracted subject-wise absolute displacement vectors (in mm), which describes the amount of movement in all directions over the whole scan as a marker of gross motion. Also, relative displacement vectors were extracted, as a marker of motion between each EPI volume. Values represent mean±SD.
